# Conventional versus minimally invasive extracorporeal circulation in patients undergoing cardiac surgery: protocol for a randomised controlled trial (COMICS)

**DOI:** 10.1177/0267659120946731

**Published:** 2020-08-12

**Keywords:** cardiac surgery, extracorporeal circulation, cardiopulmonary bypass, randomised controlled trial

## Abstract

**Introduction:**

Despite low mortality, cardiac surgery patients may experience serious life-threatening post-operative complications, often due to extracorporeal circulation and reperfusion. Miniaturised cardiopulmonary bypass (minimally invasive extracorporeal circulation) has been developed aiming to reduce the risk of post-operative complications arising with conventional extracorporeal circulation.

**Methods:**

The COMICS trial is a multi-centre, international, two-group parallel randomised controlled trial testing whether type II, III or IV minimally invasive extracorporeal circulation is effective and cost-effective compared to conventional extracorporeal circulation in patients undergoing elective or urgent coronary artery bypass grafting, aortic valve replacement or coronary artery bypass grafting + aortic valve replacement. Randomisation (1:1 ratio) is concealed and stratified by centre and surgical procedure. The primary outcome is a composite of 12 serious complications, objectively defined or adjudicated, 30 days after surgery. Secondary outcomes (at 30 days) include other serious adverse events (primary safety outcome), use of blood products, length of intensive care and hospital stay and generic health status (also at 90 days).

**Status of the trial:**

Two centres started recruiting on 08 May 2018; 10 are currently recruiting and 603 patients have been randomised (11 May 2020). The recruitment rate from 01 April 2019 to 31 March 2020 was 40-50 patients/month. About 80% have had coronary artery bypass grafting only. Adherence to allocation is good.

**Conclusions:**

The trial is feasible but criteria for progressing to a full trial were not met on time. The Trial Steering and Data Monitoring Committees have recommended that the trial should currently continue.

## Introduction

### Trial background

Despite decreasing mortality over the past decade, cardiac surgery patients may experience significant post-operative morbidity. Morbidity occurs because surgery itself carries a risk of iatrogenic harm, primarily as a result of ischaemia reperfusion injury (IRI)^1^ and the systemic inflammatory response (SIR).^2^ Although several strategies have been developed to reduce IRI and SIR (e.g. minimising the effects of perfusion and ‘conditioning’ the heart to make it more resistant to injury),^3^ these harms of surgery are often responsible for potentially life-threatening post-operative complications and delays in discharge from hospital.

Cardiac surgery with conventional extracorporeal circulation (CECC) provokes a vigorous SIR due to activation of stress pathways associated with post-operative end-organ complications (e.g. heart failure, renal impairment and neurological dysfunction). SIR is triggered by operative surgical trauma and IRI but is further exacerbated by the interaction of air, blood and synthetic components in the CECC apparatus. Minimally invasive extracorporeal circulation (MiECC) systems integrate contemporary innovations in CPB technology into a strategy which aims to reduce SIR and improve end-organ protection during cardiac surgery. They comprise a closed circuit with biologically inert blood contact surfaces and reduced priming volume; a centrifugal pump; a membrane oxygenator; a heat exchanger; a venous bubble trap or venous air removing device; a cardioplegia system and a shed-blood management device.^4^ Type IV hybrid modular MiECC systems (integrating a hard-shell venous reservoir as a stand-by component for immediate conversion to an open system) overcome safety concerns and unexpected intraoperative perfusion scenarios; thus, they are compatible with every cardiac surgical procedure.^5^

Four meta-analyses^6–9^ (with overlapping included RCTs; one also compared MiECC with off-pump coronary artery bypass grafting^9^ (CABG)) have reported substantial benefits of MIECC for 30-day mortality, stroke, myocardial infarction (MI), post-operative atrial fibrillation and renal dysfunction. No harms were reported. The diversity of MiECC systems, patients and outcomes, and the poor methodological quality of many of the randomised clinical trials (RCTs) included in these reviews, undermines the strength of this evidence and most hospitals continue to use CECC.^9^ Nevertheless, the potential benefit of MiECC highlights the urgent need to evaluate this technology in a large, high-quality RCT. The proposed trial will evaluate MiECC systems that conform to an established typology,^5^ recruit patients having the most common cardiac surgery operations, report important clinical outcomes and includes features to minimise bias.

## Methods

### Trial design and population

**Figure 1.**
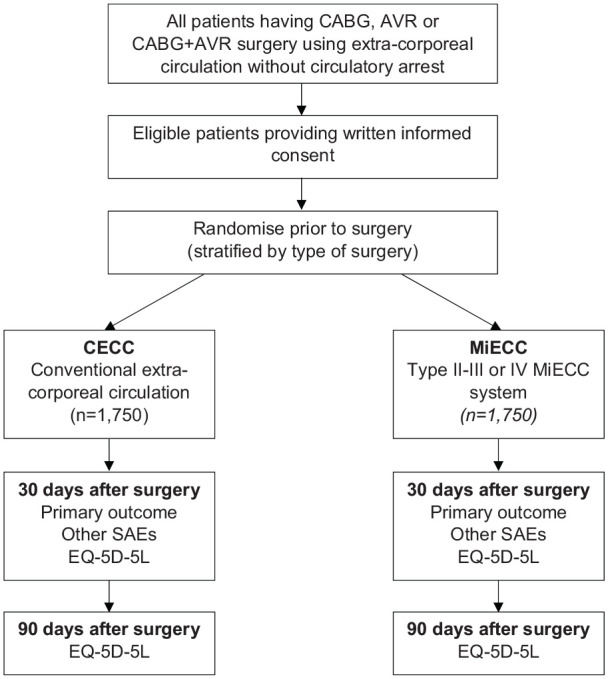
Trial schema. CABG: coronary artery bypass grafting; AVR: aortic valve replacement; CECC: conventional extracorporeal circulation; MiECC: minimally invasive extracorporeal circulation; SAE: serious adverse event.

This COMICS trial is an international, multi-centre, randomised, controlled parallel group trial to investigate the effects of using MiECC in all patients having elective or urgent coronary artery bypass grafting (CABG), aortic valve replacement (AVR) or CABG + AVR using extracorporeal circulation without circulatory arrest (Figure 1). The trial aims to test the hypothesis that MiECC is effective and cost-effective compared to CECC for most cardiac surgery operations requiring extracorporeal circulation without circulatory arrest. The trial includes a pilot and main trial phase.

The trial has three specific objectives:

1. To estimate the difference between groups in the proportion of participants experiencing the primary outcome (a composite of serious complications) up to 30 days after randomisation.
2. To compare secondary outcomes between groups: serious adverse events (SAE; events which result in death, are life threatening, require hospitalisation or prolongation of hospitalisation, result in persistent or significant disability or incapacity) not included in the primary outcome, red blood cells (RBC) and other blood products transfused; duration of cardiac ICU and hospital stay following the index admission; resource use, generic health status.
3. To estimate the cost-effectiveness of MiECC compared to CECC.

### Eligibility criteria

The trial is recruiting patients undergoing CABG, AVR or CABG + AVR. Cardiac surgery centres in Germany, Greece, Italy, Saudi Arabia, Switzerland, Turkey and the United Kingdom are participating. Additional centres in the United Kingdom, Germany and Canada are in set-up.

### Inclusion criteria

Patients are eligible if all the following apply:

1. Age ⩾18 and <85 years.
2. Undergoing any elective or urgent CABG, AVR or CABG + AVR surgery, using extracorporeal circulation without circulatory arrest.

### Exclusion criteria

Patients are ineligible if any of the following apply:

1. Requirement for emergency or salvage operation.
2. Requirement for major aortic surgery (e.g. aortic root replacement).
3. 3 Contraindication or objection (e.g. Jehovah’s Witnesses) to transfusion of blood products.
4. Congenital or acquired platelet, RBC, or clotting disorders (patients with iron deficient anaemia only are not excluded).
5. Inability to give informed consent for the trial (e.g. learning or language difficulties).

### Patient approach and consent

Potential trial participants are identified before their surgery from clinic lists and inpatient referrals. All potential participants receive an invitation letter and a patient information leaflet (PIL) describing the trial and are given time to discuss their participation with others outside the research team (e.g. relatives or friends) if they wish. Before their operation, patients are seen by a member of the local research team answers any questions, confirms eligibility and takes written informed consent if the patient is willing.

### Trial interventions

#### Minimally invasive extracorporeal circulation (MiECC; experimental intervention)

MiECC systems have evolved in a modular fashion, to address safety, volume and blood management issues. Systems have been classified according to their features (Types 1, II, III and IV).^4^ Centres may use any MiECC circuit which uses CE-marked components (or the country-specific required standards outside the European Community) and which have features consistent with Type II, III or IV criteria.

#### Conventional extracorporeal circulation (CECC; comparator intervention)

CECC should comprise (required components) standard oxygenator, roller pump, hard-cell reservoir, arterial filter, shed-blood suctions, any of a range of venting options, uncoated tubing and a cell-saver device. The following optional/alternative components can be integrated (and recorded accordingly): coated oxygenator, coated tubing and centrifugal pump. The following components are prohibited: soft-cell reservoir and vacuum-assisted venous drainage (these are advanced components which make CECC resemble a custom-made MiECC circuit).

#### Aspects of surgery common to both MiECC and CECC

Other aspects of the operations may vary by operation and centre but must be used consistently in the MiECC and CECC groups. For example, surgeons may use different cardioplegia solutions at different temperatures in different centres or for different operations. However, a surgeon carrying out one type of operation in one centre, for example, CABG in Centre A, must use the same cardioplegia solution for both MiECC and CECC. We are collecting operative details to characterise and report these variations. We believe such diversity in practice in the trial will create greater confidence in the applicability of the findings to a potential user’s clinical setting.

### Randomisation

Randomisation is performed using a secure Internet-based randomisation system, (Sealed Envelope) and is stratified by centre and surgical procedure (i.e. CABG, AVR, CABG + AVR). Participants are allocated in a 1:1 ratio to either MiECC or CECC. Randomisation takes place as close to surgery as possible and is performed by an authorised member of the local research team. Information to identify a participant and to confirm eligibility must be entered before a number is assigned (i.e. concealed randomisation).

### Blinding and other features to minimise bias

The trial includes features designed to minimise bias.^10^ Participants are blinded to the allocation. Documentary evidence is being sought wherever possible for events that qualify for the primary outcome; other events will be adjudicated. The 30-day primary outcome minimises loss to follow-up. Reporting bias will be minimised by a statistical analysis plan finalised before the database is locked.

### Trial outcomes

#### Primary outcome

The primary outcome is a composite of post-operative SAEs occurring up to 30 days after randomisation following the index admission. All SAEs that qualify for the primary outcome are objectively defined or will be adjudicated. The following events qualify:

(a) All-cause mortality up to 30 days after surgery
(b) Myocardial infarction (MI)
(c) Permanent stroke
(d) Gut infarction
(e) AKI (Acute kidney injury–an acute increase in serum creatinine > 26.4 μmol/L or a percentage increase in serum creatinine of more than or equal to 50%) Network criteria for stage 3 AKI^11^
(f) Reintubation
(g) Tracheostomy
(h) Mechanical ventilation for >48 hours, including multiple episodes when separated by more than 12 hours
(i) Reoperation
(j) Percutaneous intervention
(k) Sternal wound infection with dehiscence
(l) Septicaemia confirmed by microbiology

Events will be documented as follows: suspected MI by serum troponin concentrations and electrocardiograph recording (ECG); stroke from brain imaging (CT or MRI) report and record of new onset focal or generalised neurological deficit; gut infraction by laparotomy or post-mortem; other events from the medical record. Equivocal events will be adjudicated.

Secondary outcomes are as follows:

(a) All-cause mortality up to 30 days after surgery
(b) Other SAEs up to 30 days after surgery (primary safety outcome)
(c) Units of RBC transfused up to 30 days after surgery
(d) Other blood products transfused up to 30 days after surgery
(e) Time to discharge from cardiac ICU during the index admission
(f) Time to discharge from hospital following the index admission
(g) Delirium in ICU, assessed with the Intensive Care Delirium Screening Checklist (ICDSC)^12^ for up to 5 days (only collected at some centres).
(h) Generic health status using the EuroQol 5-level health status questionnaire (EQ-5D-5L)^13^ at 30 and 90 days after randomisation
(i) Health resources and associated costs up to 90 days after randomisation (UK centres only)

### Data collection

Participants are followed up twice, at 30 days and 90 days after surgery. Questions elicit information about SAEs (including readmissions) experienced since discharge up to 30 days after the date of surgery. Generic health status is assessed around both 30 and 90 days. The schedule of data collection is outlined in Table 1. Data are collected from participants or their medical records, entered into a bespoke database and stored on a secure server.

**Table 1.** 

	T1	T2	T3	T4	T5
Date PIS sent, date approached, age, sex, type of procedure	✓				
Eligibility check, reason for ineligibility		✓			
Consent, reason for declining		✓			
Baseline data collection		✓			
Randomisation details		✓			
Primary outcome events			✓	✓	
Blood products transfused			✓	✓	
Serious adverse events			✓	✓	
Generic health status (EQ-5D-5L)		✓		✓	✓

T1: Pre-consent (anonymised). T2: Pre-admission clinic/day before operation.

T3: During index admission. T4: 30 days. T5: 90 days.

### Sample size

The trial is powered for the primary composite outcome. The transfusion indication threshold reduction 2 trial (TITRe2) trial, carried out in 17 UK centres, showed a rate of 15-18% in the post-operative SAEs qualifying for the primary outcome in participants receiving CECC, depending on the type of surgery received (CABG, AVR, CABG + AVR).^14^ Pooled estimates from previous meta-analyses of the effects of MiECC versus CECC have reported benefits of MiECC for a range of clinical outcomes: odds ratios for 30-day mortality, 0.58^6^ 0.40^7^ 0.46;^9^ for stroke, 0.25^6^ 0.40^7^ 0.40;^9^ for MI, 0.33,^7^ 0.40;^9^ for post-operative atrial fibrillation, 0.62;^9^ renal dysfunction 0.47.^9^ A smaller but still clinically important target relative difference, that is, risk ratio of 0.75, was chosen for the trial. In order to detect a risk ratio of ⩽0.75 with 90% power and 5% significance (two-tailed), 2,504 to 3,258 participants are required, depending on the numbers recruited to different surgical strata. We propose to recruit 3,500 participants to allow for uncertainty in the assumptions underpinning this calculation.

### SAEs

SAEs are considered in three categories: SAEs that qualify for the primary outcome, other SAEs that are known complications of cardiac surgery, and SAEs that are not known complications of cardiac surgery. The latter will be subject to expedited reporting to the Lead Coordinating Centre. Expedited reporting will also include all fatal events.

### Governance

UK sites have ethics approval through the UK Integrated Research Application System (222991). Non-UK sites or countries are responsible for following their own local governance processes and obtaining the necessary approvals. The University of Bristol is acting as the sponsor for UK sites and as the central coordinating centre for non-UK sites.

### Statistical analysis

Statistical analysis is the responsibility of the Bristol Trials Centre (BTC). A final statistical analysis plan will be written, consistent with the CONSORT guidelines, including the extension for non-pharmacologic treatment interventions,^15^ and signed off before any analysis takes place.

Analyses of all outcomes apart from the primary safety outcome will be conducted according to the intention-to-treat (ITT) principle, with participants analysed according to the groups to which they were randomised. A per-protocol (PP) analysis, only including participants who received the intervention to which they were allocated, will be considered for the primary outcome if there are a considerable number of protocol deviators. The safety population will consist of all participants, where participants will be included according to the intervention they received.

#### Primary outcome analysis

The primary outcome will be assessed using a generalised linear model (GLM) to compare trial arms in terms of the risk ratio of experiencing a qualifying SAE up to 30 days after surgery, adjusting for centre and operative procedure.

#### Secondary outcome analyses

Binary secondary outcomes (all-cause mortality at 30 days after surgery; any other SAE at 30 days after surgery; any RBC transfusion; any platelet transfusion; any fresh frozen plasma transfusion) will be assessed separately, using GLMs as for the primary outcome. The total number of units of blood products transfused will also be analysed using linear regression, adjusting for centre and operative procedure, for all blood products.

Time to death, time to discharge from CICU and time to discharge from hospital will be assessed using Cox proportional hazards models to compare the trial arms, adjusting for centre and operative procedure, for both analyses. Median survival with 95% confidence intervals will be estimated by trial arm and overall for both analyses.

Generic health status (EQ-5D-5L) will be analysed using a linear mixed effects model. Mean QoL scores and 95% CIs will be summarised at baseline, 30 days and 90 days.

Occurrence and frequency of all safety events (SAEs that do not qualify for the primary outcome) will be summarised by trial arm and overall.

#### Subgroup analyses

Subgroup analyses will be carried out for the primary outcome to compare the trial arms across each level of the following characteristics:

- Age (dichotomised by the median)
- Sex (male, female)
- Operation type (CABG, AVR and CABG + AVR)
- EuroSCORE (dichotomised by the median)
- Preoperative renal dysfunction (dichotomised by the median)
- Preoperative Hb (dichotomised by the median)

All subgroup analyses will be interpreted with caution and treated as hypothesis-generating.

#### Cost-effectiveness analysis

The cost-effectiveness of MiECC compared to CECC will be estimated for COMICS participants recruited in England (approximately 600 participants), using quality-adjusted life years as the outcome measure estimated using the EQ-5D-5L, which can be mapped on to ‘valuations’.^16^

Costs of resource use for English participants will be estimated using linked Hospital Episode Statistics (HES) data and information from a previous trial.^13^ We will calculate average costs and outcomes associated with MiECC and CECC and produce an incremental cost per quality adjusted life year (QALY) gained with MiECC compared to CECC. If there are differences in mortality between trial arms, then costs and outcomes will be extrapolated to a longer time horizon. Scenario analyses will use EQ-5D-5L data from all participants and cost data from English participants.

### Status of the trial

Two centres started to recruit on 08 May 2018. Ten centres are currently recruiting; another centre recruited for 14 months but stopped participating for reasons unrelated to the trial. About 40-50 patients were randomised per month from 01 April 2019 to 31 March 2020 with over 600 now randomised. About 80% of participants have had CABG only. Primary outcomes are available in the database for 85%, recognising that these may still have to be entered for recent recruits. Adherence to randomised allocation is good (99%).

## Discussion/challenges

There have been significant challenges in setting up the trial and achieving the rate of recruitment required to complete the trial in a feasible duration (scheduled to recruit for 3 years from September 2020). A joint meeting of the Trial Steering and Data Monitoring Committees reviewed progress against criteria for continuing to the main trial in December 2019, 18 months after starting the pilot: ⩾16 centres recruiting; ⩾750 participants randomised; >90% adherence to the allocated intervention. These criteria were not met, primarily due to fewer than expected centres joining the trial especially during 2018. Nevertheless, the Committee chairs recommended continuing for a further 6-9 months, aiming to increase the recruitment rate and obtaining funding for the main trial. Additional centres are currently being set up and an application for funding has been submitted. New centres are welcome to join the trial (please email the contact address provided for further details).

Negotiating contracts with each participating site has been the most serious challenge. The contract describes indemnity arrangements, key responsibilities of different parties, intellectual property and data ownership, data sharing and publication policy, even if no funding is offered. The main obstacles have been indemnity (the coordinating centre is not providing any), intellectual property ownership (owned by the coordinating centre) and the legal system under which any disputes would be considered (both governing law and jurisdiction), which have prevented some centres from taking part.

Some centres have been unable to take part because there is no funding yet to reimburse their research costs; one potential centre considered this unethical. Award of funding would address this challenge and potentially increase recruitment in centres already recruiting.

The capital cost of adopting MiECC has been a barrier for centres wanting to use it. Adopting MiECC also requires entire surgical teams (surgeon, anaesthetist and perfusion team) to agree. MiECC also has higher consumable costs, although these may potentially be offset by clinical benefits (see above). European guidelines recommend that MiECC should be considered instead of CECC to increase the biocompatibility of extracorporeal circulation technology used in cardiac surgery (class of recommendation IIA, level of evidence B).^17^

The COMICS trial is already the largest randomised trial of MiECC compared to CECC in patients having most cardiac surgery operations requiring extracorporeal circulation without circulatory arrest. The COMICS trial will have substantial health and socioeconomic impacts. It will quantify the effects of MiECC versus CECC on a range of important clinical outcomes (major post-operative complications, mortality, etc.), health resource use (blood product use, intensive care unit and hospital stay, etc.), health-related quality of life (EQ-5D-5L) and QALYs.

## Appendix 1

### The COMICS investigators

Gianni D Angelini, University of Bristol; Barnaby C Reeves, University of Bristol; Jonathan Evans, University of Bristol; Lucy A Culliford, University of Bristol; Laura Collett, University of Bristol; Chris A Rogers, University of Bristol; Elizabeth Stokes, University of Oxford; Kyriakos Anastasiadis, AHEPA University Hospital; Polychronis Antonitsis, AHEPA University Hospital; Thierry Carrel, University Hospital Bern; Dorothée Keller, University Hospital Bern; Andreas Liebold, Universitätsklinikum Ulm; Fatma Ashkanani Universitätsklinikum Ulm; Aschraf El-Essawi, Universitätsmedizin Göttingen; Ingo Breitenbach, Klinikum Braunschweig; Clinton Lloyd, University Hospitals Plymouth NHS Trust; Mark Bennett, University Hospitals Plymouth NHS Trust; Alex Cale, Hull University Teaching Hospitals NHS Trust; Lindsay Mclean, Hull University Teaching Hospitals NHS Trust; Serdar Gunaydin, Numune Training and Research Hospital in Ankara; Eren Gunertem, Numune Training and Research Hospital in Ankara; Farouk Oueida, Saud Al-Babtain Cardiac Centre; Ibrahim Yassin, Saud Al-Babtain Cardiac Centre; Cyril Serrick, University Health Network Toronto; Vivek Rao, University Health Network Toronto; Marco Moscarelli, Anthea Hospital; Bari, Ignazio Condello; Anthea Hospital, Bari; Prakash Punjabi, Imperial College Healthcare; Cha Rajakaruna, University Hospitals Bristol NHS Foundation Trust; Daniel Bone, University Hospitals Bristol NHS Foundation Trust; William Lansdown, University Hospitals Bristol NHS Foundation Trust; Narain Moorjani, Royal Papworth Hospital; Sarah Dennis, Royal Papworth Hospital.
